# Aging Chart: a community resource for rapid exploratory pathway analysis of age-related processes

**DOI:** 10.1093/nar/gkv1287

**Published:** 2015-11-23

**Authors:** Alexey Moskalev, Svetlana Zhikrivetskaya, Mikhail Shaposhnikov, Evgenia Dobrovolskaya, Roman Gurinovich, Oleg Kuryan, Aleksandr Pashuk, Leslie C. Jellen, Alex Aliper, Alex Peregudov, Alex Zhavoronkov

**Affiliations:** 1 Laboratory of molecular radiobiology and gerontology, Institute of Biology of Komi Science Center of Ural Branch of Russian Academy of Sciences, Syktyvkar, 167982, Russia; 2 Laboratory of genetics of aging and longevity, Moscow Institute of Physics and Technology, Dolgoprudny, 141700, Russia; 3 Laboratory of postgenomic studies, Engelhardt Institute of Molecular Biology of Russian Academy of Sciences, Moscow, 119991, Russia; 4 School of Systems Biology, George Mason University, VA, Manassas, 20110, USA; 5 Branch of N.I.Pirogov Russian State Medical University "Scientific Clinical Center of Gerontology", Moscow, 117997, Russia; 6 Xpansa, Conzl OU, Mustamae Tee 5, Tallinn, 10616, Estonia; 7 Infinity Sciences, Inc, 16192 Coastal Highway, Lewes, Delaware, County of Sussex, 19958, USA; 8 Genetics, Genomics, and Informatics, University of Tennessee Health Science Center, Memphis, TN, 38163, USA; 9 D.Rogachev FRC Center for Pediatric Hematology, Oncology and Immunology, Samory Machela 1, Moscow, 117997, Russia; 10 Insilico Medicine, Inc, Johns Hopkins University, ETC, B310, Baltimore, MD, 21218, USA; 11 The Biogerontology Research Foundation, 2354 Chynoweth House, Trevissome Park, Blackwater, Truro, Cornwall TR4 8UN, UK

## Abstract

Aging research is a multi-disciplinary field encompassing knowledge from many areas of basic, applied and clinical research. Age-related processes occur on molecular, cellular, tissue, organ, system, organismal and even psychological levels, trigger the onset of multiple debilitating diseases and lead to a loss of function, and there is a need for a unified knowledge repository designed to track, analyze and visualize the cause and effect relationships and interactions between the many elements and processes on all levels. Aging Chart (http://agingchart.org/) is a new, community-curated collection of aging pathways and knowledge that provides a platform for rapid exploratory analysis. Building on an initial content base constructed by a team of experts from peer-reviewed literature, users can integrate new data into biological pathway diagrams for a visible, intuitive, top-down framework of aging processes that fosters knowledge-building and collaboration. As the body of knowledge in aging research is rapidly increasing, an open visual encyclopedia of aging processes will be useful to both the new entrants and experts in the field.

## INTRODUCTION

As the world population is rapidly aging, the prevalence of aging-related diseases and the demand for expensive, long term health care is also rising (1–4). To offset the burden of this shift, scientific knowledge and innovation will become increasingly crucial, and anti-aging and disease prevention strategies will become national and international priorities. Aging research as a field will boom. Nevertheless, it faces several challenges, and the growth will need direction.

One of the challenges is the current lack of a freely available, comprehensive collection of aging-related biological pathways and encyclopedia of aging knowledge. Biological pathways are one of the most powerful visualization tools in biology (5). They provide an intuitive, systems view of the interactions between the multitude of individual elements in any given process. They can be interactive for user-directed exploration and amenable to computational methods, and they are indispensable in making sense of large-scale data sets, where a multitude of individual changes may reflect a small number of more biologically important (and more statistically powerful) changes at the pathway level (6). Pathway collections are a key feature of many biological data repositories in the public domain (7).

Aging processes are particularly suited for representation by a pathway collection. The complex series of orchestrated events and multiple biological and environmental factors that interact in aging can be best captured in a collection of top-down, systems-level biological pathways, which depict and characterize the nature of these interactions.

Characterizing aging pathways may not only help develop aging theories, but may actually lead to breakthroughs in intervention. Examples of practical applications include: (i) targeting aging-related signaling pathways in screening for anti-aging compounds, or geroprotectors (8), (ii) mimicking anti-aging intervention pathways with limited translational value (e.g. caloric restriction), pharmacologically or otherwise and (iii) comparing pathways altered in aging-related diseases to those in typical aging cells (9–12), to identify aging processes that may also be mechanisms of disease.

The lack of an aging pathway collection until now may reflect the fledgling nature of the field but also stems in part from the sheer diversity of aging-related processes. Characterizing these is a monumental task. Aging itself is a complex process that occurs at all levels in all systems of the body, leads to a loss of function and triggers a number of diseases. There is ongoing debate as to whether aging is itself a treatable disease (13). As such, aging research involves a highly diverse community of researchers with various perspectives.

If any single narrative of aging mechanisms is to be constructed, the community needs a platform where knowledge can be pieced together collaboratively into pathways, node by node, and ultimately into a unified theory.

There have been many previous attempts at structuring aging data and knowledge on the web (14–19), but there is still a need for a rapid, intuitive, visual overview of aging processes, from environmental triggers down to molecular interactions. To our knowledge, no such resource yet exists. To fill this gap, we have developed Aging Chart, a wiki-based, community-curated biological pathway collection and encyclopedia of aging processes. Aging Chart will complement and add to the existing set of public aging-related data- and knowledge bases on the web.

### The community curation model

Pathway collections are only as useful as they are accurate. As new data stream in, pathway content must be maintained. Pathway content is not readily accessed from raw data and must be manually integrated from the literature in most cases, with each individual relationship between elements validated in a peer-reviewed study. In the past, this was performed by small teams of experts; however, as the rate of new data outpaced their efforts, it was difficult to prevent centrally-curated pathway collections from becoming outdated and incomplete (7). Ultimately, enlistment of the community in this effort was suggested, and community collaboration and curation of pathways became the rising model in response to this problem (7,20,21).

WikiPathways, introduced in 2008, was the first successful attempt at a community-curated model for pathway collections (20). Following the Wikipedia model (http://www.wikipedia.org/), it was developed as a wiki-based, open-source platform designed to be used with utmost ease, keeping barriers low for non-technical users who wish to contribute pathway content. It has been highly successful (21), with an active audience of readers and contributors. Its content is of publishable quality, supporting community collaboration as an enhancement of quality control, not *vice versa*. Wikis have shown to be useful for other biological applications, as well, such as the Gene Wiki (22), a portal within Wikipedia, and Wikiproteins, a community annotation resource (23).

The scope is of WikiPathways is broad, encompassing any field of biology and any organism of interest, but it also now hosts a number of portals that link earlier collections and other, more specific pathway collections that appeal to prominent subtopics (e.g. Reactome (24), Micronutrients (25)). The development and ongoing success of these collections shows demand for area-specific collections of pathways and data and suggests that a community-curated pathway collection for Aging would similarly thrive; there are several reasons for this. First, the smaller audience may be balanced by greater contributor expertise. Second, smaller, more specific pathways may be preferable to so-called hairball pathways of larger scope. Third, specific collections offer a succinct overview for scientists with tangential lines of research but limited time, thus may promote growth in relatively new or highly multidisciplinary areas, such as aging. Finally, area-specific pathway collections can promote community-building and collaboration.

Presently, there is no specific portal providing access to interconnected pathways strongly implicated in mechanisms of aging and longevity on WikiPathways or anywhere else. Aging Chart is the first attempt to build such a collection.

### Aging Chart: a community-curated collection of pathways on aging processes

In response to the growing need for an aging pathway collection to consolidate, integrate and provide a visual overview of the vast array of knowledge available to date on aging processes, we created Aging Chart.

Following the community collaboration model, Aging Chart is a complex, wiki-based system designed to reign in and redirect the horizontal growth of the aging field by centralizing data on aging processes into one pathway-oriented, community-curated site, where users can easily input and retrieve content and integrate it into a visible, intuitive, big-picture framework. Gleaned from peer-reviewed literature, Aging Chart makes its debut stocked with 114 pathways, networks and concept maps on all topics related to aging, from gene-centered pathways to those describing aging processes, age-related diseases, longevity factors and anti-aging strategies. These are all freely available for exploration and updates from users who wish to contribute. Contributions are openly encouraged. The pathway diagrams are interactive, with clickable nodes for user-led exploration that link to related pages and pathways for any particular element of interest. Concise textual descriptions of current anti-aging intervention strategies are also available, with PubMed links to peer-reviewed literature. Aging Chart was designed for the technical and non-technical user alike. It provides a platform for the rapid learning of virtually any aspect of aging that is currently understood as well as for the integration of elements and concepts that may lead to the generation of new hypotheses and research directions.

With the launch of Aging Chart, we aim to provide an open visual encyclopedia of aging processes to benefit the new entrants and experts in the field alike.

## MATERIALS AND METHODS

Aging Chart is a complex system featuring over one hundred aging-related pathways designed to counter the challenges in data management faced by the field of aging research at this time. We developed it to accomplish two overarching goals: (i) to centralize and organize the existing sprawl of scattered aging data and knowledge and (ii) to enable and encourage researchers to access and contribute to these data and knowledge. This involved four tasks of equal importance, presented below in order of implementation:

1. Integration of existing data in various formats (diagrams, text, links, etc.)
2. Organization of integrated data, while maintaining the integrity and relationships between elements
3. Creation of a user-friendly interface for non-technical users
4. Promotion of community collaboration by providing users with the option to add new information, edit existing data and view page history.

We built Aging Chart as a wiki system, using the MediaWiki application to support community involvement in the development and curation of the data. We selected MediaWiki (http://www.mediawiki.org/) among the various wikis for several reasons. First, it is fully open source, constantly updated, and has an active community. In addition, it has many useful extensions, a user management subsystem, a discussions mechanism, and is user-friendly in terms of categorization, changes in look and feel, etc.

The first stage of development in creating Aging Chart was the integration of data from different sources: diagrams in Cmap format (http://cmap.ihmc.us/), text descriptions for each diagram and links to external publications. While MediaWiki provides user-friendly support for many tasks, including text editing, creating new pages and uploading static pictures and video, it does not support Cmap format. Due to the lack of single industry specific solution and need of easy network visualization tool, scientific team of Aging Chart selected CMAP as tool for the first version of the pathways database. Application saves data in CXL format that is type of XML standard (http://cmap.ihmc.us/xml/CXL.html#properties-list). That means possibility to create conversion application to move data to PathVisio, Cytoscape and other formats. The relationship between pages and clickable nodes on diagrams were critical requirements for this project. We found a small extension for the wiki that allows use of the clickable diagrams in Graphviz format (http://www.graphviz.org/) and incorporation of these into wikipages. We then wrote a parser with a simple graphical interface that allows it to convert to special Graphviz format and to upload source diagrams to Aging Chart wiki (Supplementary Figure S1). When uploading, the user can choose the title for a new page (or title of page to update if existing) and categories for easy classification.

After uploading, the parser changes the legend to Aging Chart format with pre-defined rules. In additional, we created a bulk upload script to quickly update many pages at once.

Preliminary research has shown that the standard design is convenient for most users. With slight changes in page styles, the Aging Chart wiki took on a modern, fresh appearance. We designed the main page with Twitter Bootstrap (http://getbootstrap.com/). It includes six main blocks: a Twitter newsfeed, two main diagrams (Mechanism of Aging and Basis of Longevity), Interventions; Featured research, and Resources. The role of this page is a ‘getting started’ site for new users.

The next stage of development was choice of page structure. After considering various options, we chose the structure with the most optimal and intuitive view (Supplementary Figure S2). Page width automatically changes depending on the width of the largest diagram.

Creation of pop-ups for each node on a diagram was another important task. These elements contain:

- title of the node;
- short description for the node;
- links to related publications and diagrams that automatically load when user clicks on a node.

We used JavaScript library qTip2 (http://qtip2.com/) as a basis for pop-up windows. An automatic search of related diagrams implemented with AJAX and MediaWiki API. The user can manually add links for each node also. In the future, it will support automatic searches of similar links in Wikipedia and other resources.

Next, it was necessary to organize a user-friendly editing mode. The first version of the Aging Chart wiki required knowledge of HTML and CSS to work with content. We decided to split the standard MediaWiki editing field into separate ones for each part of a page: a diagram, two fields for each pop-up window, description and links (Supplementary Figure S3).

In addition, most of the HTML code is generated automatically without a need for user editing and is hidden from users to avoid problems and simplify work with the wiki. However, if necessary, the user can easily add a piece of HTML code.

It was important to give users the ability to update only part of the content while saving the rest. Thus, we developed Aging Chart such that the user can add small changes to a diagram without changing the mini-review and content for nodes. Our algorithm checks what changes have been made and removes unnecessary content while leaving useful information. Meanwhile, prior versions of pages are archived and available for viewing.

To protect Aging Chart Wiki from vandalism and spam, we implemented an account confirmation system. When registering, the user fills out and submits a short form and, once approved by an administrator, receives a confirmation email.

**Figure 1.**
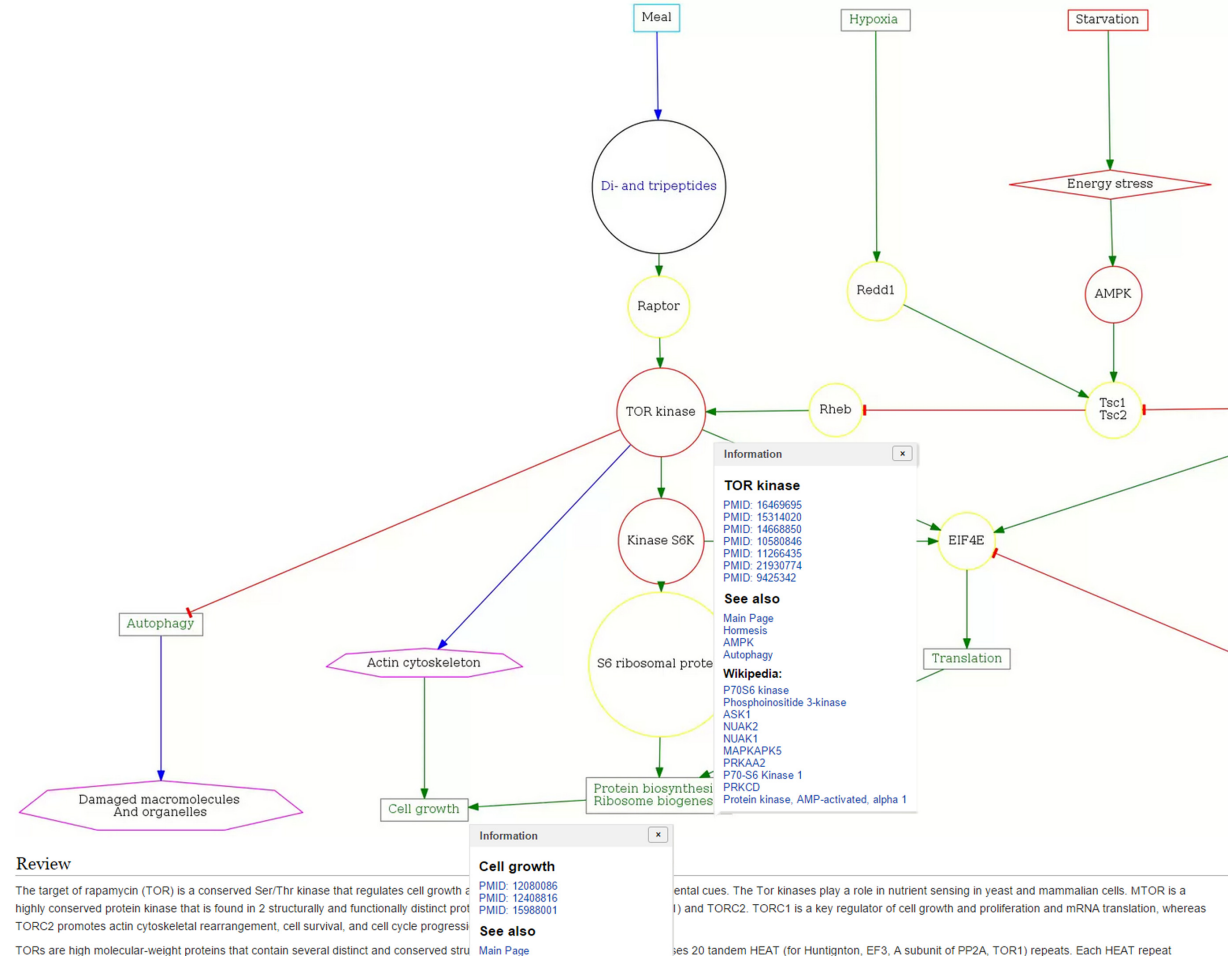
Example of the mTOR pathway presented in editable format and linked to multiple information resources. Pop-ups appear upon clicking each node with links to PubMed references and other relevant Aging Chart pathways. Mini-reviews are found at the bottom of each pathway page and summarize pathway information with current peer-reviewed literature and PubMed links.

With the basic structure of the system in place, pathway content was organized and developed. A total of 114 aging-related pathway diagrams were manually constructed. Each diagram summarizes a brief sequence of related events significant to aging, and each sequence represents a mechanism or process consisting of interactions between key elements. The target of rapamycin (TOR) kinase pathway is an illustrative example (Figure 1). Elements range from molecules (e.g. TOR) to environmental triggers (e.g. starvation). The pathway content, including each interaction between elements, was manually selected from peer-reviewed literature and referenced with links to articles on PubMed, which appear in a small pop-up window after clicking on a pathway node of interest. These can refer to the node subject itself, an interaction between nodes, or the link between the node subject and aging processes. Related pathways are also linked in this window. Additionally, each pathway is summarized with a mini-review. Each mini-review includes general definitions, underlying mechanisms, and the link to aging, and is composed using the same set of references compiled to construct the pathway.

### Future directions

The MediaWiki engine provides many features that can be implemented in the future. The next phase of the Aging Chart project will include development of an enhanced search system that will help users to find necessary information in a few clicks. This system will work with external data sources such as PubMed, KEGG, Wikipedia, etc. In addition, it will have its own database, which users can fill manually. As already mentioned before, pop-up windows will contain additional information, which will be generated automatically by searching different sources. Another major part of the second phase will be the creation of a unified online editor.

Audience and community participation will be paramount as Aging Chart is launched. While the site is stocked with over 100 pathways at the outset, establishing an active set of contributors will be crucial to the ongoing building and maintenance of the pathways on Aging Chart, and community collaboration depends on diligence of community members in ensuring that new findings are incorporated and errors or outdated findings corrected. The multidisciplinary nature of aging research demands that the contributing audience of experts be from a broad range of research areas.

Given the importance of building a community of contributors, it may appear counterintuitive that we developed Aging Chart as an independent resource, rather than a portal in WikiPathways. Wikipathways set the standard for community curation and has a well-established community of contributors, and as such an aging portal within WikiPathways would be an ultimate goal to work toward, offering the broadest audience with the most sophisticated platform for pathway curation; however, independent development of Aging Chart at the outset was more befitting for several reasons. Unlike many other areas of research, aging research is a field with many disagreements in entire theories of aging, longevity and strategies to address the pathological changes, and there is constant debate regarding whether aging is a disease (13). Established and highly functional tools specializing in pathway construction, curation and visualization like Wikipathways may not provide sufficient motivation to build a community for this field with so many opposing views. In order to consolidate and curate the opinions, there needs to be a specialized community with high-level process diagrams where most scientists are in agreement; this will lead to low-level signaling and metabolic pathways that can serve as sandbox for later integration with Wikipathways, and may possibly lead to a collaborative thematic resource, by example of a community for plants (26). Collaboration with existing aging resources would also grow the audience of expert contributors and users alike, so would also be a goal at any point in the future.

## CONCLUSION

Aging Chart is a new, wiki-based visual encyclopedia of aging processes that will facilitate rapid exploration of aging pathways and mechanisms by experts and entrants to the field alike. It is fully open source, features interactive, intuitive pathways, and encourages community curation and contribution of content. Aging Chart is an extension of the community curation model for pathway collections and is the first such resource to serve the growing community of researchers interested in the cellular and molecular biology of aging.
